# Swelling of Zein Matrix Tablets Benchmarked against HPMC and Ethylcellulose: Challenging the Matrix Performance by the Addition of Co-Excipients

**DOI:** 10.3390/pharmaceutics11100513

**Published:** 2019-10-04

**Authors:** Alberto Berardi, Safwan Abdel Rahim, Lorina Bisharat, Marco Cespi

**Affiliations:** 1 Department of Pharmaceutical Sciences and Pharmaceutics, Faculty of Pharmacy, Applied Science Private University, Amman 11931, Jordan; 2 Department of Pharmaceutics and Pharmaceutical Technology, School of Pharmacy, The University of Jordan, Amman 11942, Jordan; l.bisharat@ju.edu.jo; 3 School of Pharmacy, University of Camerino, 62032 Camerino (MC), Italy; marco.cespi@unicam.it

**Keywords:** zein, swelling, matrix tablets, USB microscope, HPMC, ethylcellulose, excipients, image analysis

## Abstract

Zein is an insoluble, yet swellable, biopolymer that has been extensively studied for its applications in drug delivery. Here, we screened the effect of co-excipients on the swelling and drug release of zein tablets. All throughout the study the behavior of zein was benchmarked against that of hydroxypropyl methylcellulose (HPMC) and ethylcellulose (EC). Tablets containing either zein, HPMC, or EC alone or in combination with co-excipients, namely lactose, dicalcium phosphate (DCP), microcrystalline cellulose (MCC), polyvinylpyrrolidone (PVP), or sodium lauryl sulfate (SLS) were prepared by direct compression. Matrix swelling was studied by taking continuous pictures of the tablets over 20 h, using a USB microscope connected to a PC. The overall size change and the axial and radial expansion of the tablets were automatically extrapolated from the pictures by image analysis. Moreover, drug release from tablets containing ternary mixtures of zein, co-excipients and 10% propranolol HCl was also studied. Results showed that zein matrices swelled rapidly at first, but then a plateau was reached, resulting in an initial rapid drug burst followed by slow drug release. HPMC tablets swelled to a greater extent and more gradually, providing a more constant drug release rate. EC did not practically swell, giving a nearly constant drug release pattern. Among the additives studied, only MCC increased the swelling of zein up to nearly three-fold, and thus suppressed drug burst from zein matrices and provided a nearly constant drug release over the test duration. Overall, the incorporation of co-excipients influenced the swelling behavior of zein to a greater extent compared to that of HPMC and EC, indicating that the molecular interactions of zein and additives are clearly more complex and distinct.

## 1. Introduction

Zein is the main storage protein of corn. In the last few years, zein has gained tremendous popularity as a novel biocompatible and biodegradable material, produced from sustainable sources. Possible pharmaceutical and biomedical applications of zein include its use in tissue engineering [1,2] and in the formulation of microparticles, nanoparticles, and films [3,4]. Moreover, zein has also been utilized as a matrix-forming polymer for controlled drug release in macroscopic pharmaceutical dosage forms [5], including tablets [6,7,8,9], capsules [10], and hot-melt extruded caplets [11,12].

The molecular interactions of zein with surrounding solvents [6], ions [10], drugs [12], and formulation additives [13] are much more complex than other pharmaceutical polymers used in oral solid dosage forms. In fact, while most pharmaceutical homo- and co-polymers have highly repetitious structures, zein is a sequence of many different monomers (i.e., amino acids), with several domains and regions, which are variable at the level of primary, secondary, and tertiary structure of the protein [14]. Due to this heterogenous and complex physico-chemical composition, the molecular interactions of zein with neighboring compounds could be distinct and unpredictable.

A significant example of this unique behavior of zein is its matrix-forming ability. In fact, zein, which is constituted of a high proportion of non-polar amino acids and only low amounts of polar amino acids [14], is an insoluble polymer. However, despite being insoluble, it can hydrate and swell in water. Therefore, the behavior of zein is unlike that of typical insoluble polymers, such as ethylcellulose (EC), that are non-swellable [15]. Zein also acts differently from hydrophilic swelling polymers, such as hydroxypropylmethylcellulose (HPMC), which are soluble and erodible [16]. Thus, zein, being swellable, yet non-erodible, can be considered an amphiphilic polymer with dual properties [5].

The use of zein in the development of solid dosage forms would require the incorporation of additives, i.e., co-excipients, which are used to either improve manufacturability of the dosage forms (e.g., diluents and binders) [7,13], and/or modulate the drug release kinetics (e.g., surfactants and pore-formers). For example, in a previous work, we showed that the flow and compaction properties of zein-based tablet formulations could be improved by the addition of silicified microcrystalline cellulose (SMCC). Moreover, drug release profiles and swelling of the tablets were also affected by the concentration of SMCC in the formulation [13]. The influence of other common formulation additives on the swelling and release kinetics of zein-based tablets is currently unknown. Thus, in this work, we studied the effect of the incorporation of common diluents (lactose (Lac), dicalcium phosphate (DCP), microcrystalline cellulose (MCC)), a binder (polyvinylpyrrolidone (PVP)) and a surfactant (sodium lauryl sulphate (SLS)) on the swelling and release kinetics of zein-based tablets.

The experimental design of this study has some unique features:

1. The swelling of zein-matrices was studied by image analysis in a nearly-continuous mode: We utilized a USB-microscope connected to a PC to automatically collect images of the swelling tablets over 20 h, at a frequency of one frame every 10 min. Image sequences were then binarized using image analysis software, so to enable the calculation of the percentages of total tablet swelling, as well as the extent of swelling in the axial and radial directions, over time. The facile image analysis method developed here, functioned as a direct readout of tablet swelling.
2. With the broader aim to underpin the unique matrix-forming ability of zein, the performance of zein tablets was compared to that of HPMC and EC tablets, as reference controls of purely hydrophilic/swellable and insoluble matrices, respectively. Indeed, all throughout the study, swelling measurements of zein/co-excipients matrices were systematically compared with those of HPMC/co-excipients and EC/co-excipients matrices.

Given the recently growing interest in biopolymers in the pharmaceutical and nutraceutical industries, zein could be considered an attractive alternative to commonly used controlled-release polymers, such as HPMC and EC. Thus, extensive evaluation of zein as a matrix-forming polymer is needed [5]. In the specific, this work will ultimately provide a database on the performance of zein in combination with the most common formulation additives. Such a database will greatly facilitate the development of functional zein-based matrix tablets. The findings of this study could also pave the path towards a comprehensive understanding of the complex physico-chemical behaviour and molecular interactions of zein in matrix tablets.

## 2. Materials and Methods

### 2.1. Materials

Zein was purchased from Sigma (Z3625, with a reported protein content of 86.06% *w*/*w* [17] and molecular weight primarily of 22–24 kDa [18], corresponding to α-zein [19]). For the complete protein analysis of zein (Sigma Z3625) by sodium dodecyl sulfate polyacrylamide gel electrophoresis readers are referred to [20]. Hydroxypropyl methylcellulose (HPMC-Methocel^®^ K4M Premium CR, i.e., premium grade cellulose ether type 2208, 4000 cps [21]) and ethyl cellulose (EC—Ethocel Standard 10 Premium, i.e., premium grade cellulose ethyl ether, 10 cps [21]) were provided by Colorcon and The Dow Chemical Company, respectively. Lactose monohydrate (Foremost #316 Fast Flo NF) and Polyvinylpyrrolidone (PVP) K30 were supplied by Foremost Farms and Shanghai Yuking Water Soluble Material Tech Co., respectively. Sodium lauryl sulphate was bought from Acros Organics. Titanium dioxide was obtained from Xilong Scientific Co. and magnesium stearate from Laboratory Rasayan. Dibasic calcium phosphate anhydrous (DCP, Emcompress-JRS Pharma) and microcrystalline cellulose (MCC—Vivapur^®^ 102) were gifted by Rimon Chemical Co. Propranolol HCl was generously donated by Hikma Pharmaceuticals.

### 2.2. Methods

#### 2.2.1. Preparation of Tablets

Tablets containing polymers (i.e., zein, HPMC, and EC) only, binary mixtures of polymers and propranolol HCl, or binary mixtures of polymers and various co-excipients each at high and low concentrations were prepared. Moreover, ternary mixtures containing zein, co-excipients and propranolol HCl were also formulated. The composition of zein-, HPMC-, EC-based formulations are presented in Table 1, Table 2 and Table 3, respectively.

It can be noticed that the ratios of zein/co-excipients in the binary mixtures were maintained the same in the corresponding formulations of the ternary mixtures (Table 1). Moreover, as shown in Table 2, 0.5% TiO_2_ (insoluble, white pigment) was added to all HPMC formulations in order to render formed gels of HPMC tablets opaque and thus facilitate measurements by image analysis.

Tablets of 600 mg were prepared by direct compression using a manual press (Riken Seiki, Ojiya, Japan), equipped with 13-mm round flat-faced punches. Tablet hardness was maintained between 150 and 180 N for tablets made of pure polymers, and between 120 and 170 N for all other formulations. The variability in hardness, although minimal, was unavoidable due to the challenge of compressing materials with different compaction properties. For instance, zein has poor tabletability, unlike HPMC and EC [13,22].

#### 2.2.2. Swelling Studies

The volumetric swelling of tablets was measured by taking pictures of the tablets using a USB-microscope and analyzing the collected images with an image analysis software. A technique previously developed to study the disintegration of immediate-release tablets was adapted here to study tablet swelling [23,24,25].

Briefly, a beaker (14 cm diameter) was positioned over a LED white light source. A USB-microscope (Plugable 250× Digital USB Microscope, Plugable Technologies, Redmond, WA, USA) connected to a computer was positioned at a fixed height over the beaker. A tablet was then placed on its side onto the bottom of the beaker and fixed with a micro-drop of cyanoacrylate adhesive glue. First, an image of the dry tablet, corresponding to time 0, was taken by the USB-microscope. Then 300 mL of 50 mM phosphate buffer of pH 6.8 was poured into the beaker. Images of the swelling tablets were collected at a frequency of one image every 10 min for 20 consecutive hours. Experiments were conducted in triplicate.

The image sequence of a swelling tablet was analyzed using ImageJ software (1.49 v; National Institutes of Health). The tablet appeared as dark “particle” (i.e., rectangle) on a white background. Stacks of the 121 images of the same tablet during the swelling were binarized using the “default” setting. Then, the “area” and the “fit ellipse” of the “particle” in each image were calculated, providing indications about the projected area and axial and radial dimensions of the tablet. Data were finally presented as tablet size change (i.e., change in the projected area) as a function of time, and as radial and axial expansion as a function of time. For a qualitative presentation of swelling process, images of the same tablet at time points 0, 10 min and 1, 3, 8 and 20 h were selected, binarized and presented as a Z-stack (i.e., overlapped frames).

#### 2.2.3. Drug Release Studies

Drug release from the tablets was studied using a USP dissolution apparatus I (Pharma Test PTW 2, Hainburg, Germany) at 37.0 ± 0.5 °C. 900 mL of 50 mM phosphate buffer of pH 6.8 was used as dissolution media. Tests were conducted for 6 h at a rotation speed of 100 rpm. Drug release was measured spectrophotometrically at 289 nm (Milton Roy 601, Rochester, NY, USA). Each study was performed in triplicate. Results were plotted as the mean percentage of drug release as a function of time. Moreover, the initial drug burst from each formulation, calculated as the average dissolution rate in the first 10 min of exposure to fluids, was presented.

It should be noted that a single medium, i.e., phosphate buffer, was used in the swelling and release studies, in order to minimize the number of confounding factors in the discussion of the influence of excipients on zein. Nevertheless, the effect of medium pH and ionic strength on zein matrices has been previously well characterized [5,7,10,11,12,13].

## 3. Results and Discussion

### 3.1. Swelling and Drug Release of Matrices of Pure Polymers

**Figure 1:**
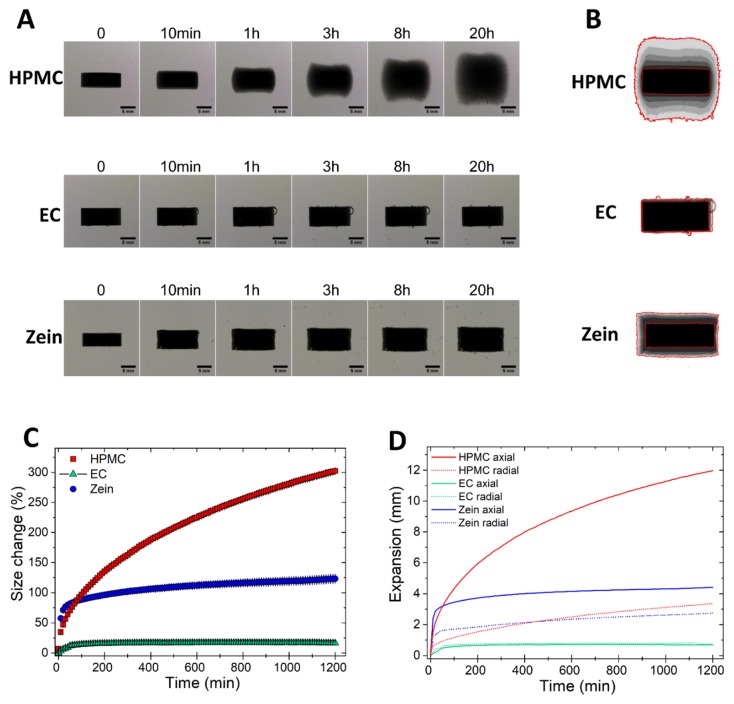
Swelling of tablets of pure HPMC, EC, and zein. (**A**) Image sequence of tablet swelling. (**B**) Sum of Z-stack of images. The two red lines are the outlines of the tablet at time 0 and at 20 h. (**C**) Swelling measured as the change of the projected area of tablets (mean ± SD, *n* = 3) as a function of time. (**D**) Axial and radial swelling of tablets (mean ± SD, *n* = 3) as a function of time.

The swelling of compacts made of pure HPMC, EC, and zein was first determined using the imaging technique described earlier. In video S1, a time-lapse shows the morphological changes occurring to HPMC (left), EC (center), and zein (right) tablets exposed to aqueous media for over 20 h (1 s in the video = 40 min). Figure 1A,B presents selected images and Z-stack (i.e., overlapped frames) of the same tablets at different time points. The collected images (121 for each tablet over the 20-h test) were used for the quantitative calculation of the change in the projected area of the tablets during swelling (Figure 1C), as well as their expansion in the axial and radial directions (Figure 1D).

Figure 1 shows that the hydrophilic HPMC had more than 300% increase in the projected area over the 20 h of exposure to fluids. The swelling was gradual, due to the rapid formation of a viscous gel on the tablet surface, which enabled slow hydration of the core [16]. The expansion of HPMC was mainly in the axial direction (Figure 1A,B,D), in agreement with previous findings [26,27,28,29,30]. The preferentially axial swelling is not due to the gelling of HPMC, which is omnidirectional, but to the axial relaxation of the dry core [26,27]. On the contrary, EC, being an insoluble and relatively hydrophobic polymer, had a negligible expansion in both directions (Figure 1A–D) [31]. Zein swelled rapidly in the first minutes of exposure to fluids, but then the size expansion nearly reached a plateau with 123 (±8) % increase in projected area over the 20-h test (Figure 1A–C). The extent of radial swelling of zein compacts was similar to that of HPMC, while the axial expansion was much lower (Figure 1D).

The kinetics of swelling of zein, a hydrophobic yet swellable polymer [5], are unlike those of both the hydrophilic HPMC and the hydrophobic EC. Zein initially swells more rapidly than HPMC (Figure 1C). This is because, while HPMC gels immediately at the surface, hindering further hydration of the core, zein takes more time to hydrate at the tablet surface, closing-up the voids between particles and creating a barrier to water penetration. Thus, more water can initially infiltrate through zein tablets, favoring the swelling of zein. However, once the polymer swells, it forms a barrier and slows down further water ingress to the core. This is evident by the minimal swelling of zein compacts at times greater than 200 min.

The direction of the volumetric expansion is nearly omni-directional for zein and mainly axial for HPMC. This can probably be ascribed to the different compaction behavior of the two materials. Having good compaction properties and undergoing plastic deformation upon compression [32,33], HPMC expands mainly axially upon exposure to fluids, due to plasticization by water molecules and the consequent release of the stored energy of compression. On the contrary, zein, which has poor tabletability [13], is not likely to undergo significant plastic deformation, and thus, swells in all directions. A similar correlation between compaction properties and mechanism of tablets expansion has also been found in immediate-release tablets containing swelling disintegrants [23,25].

**Figure 2:**
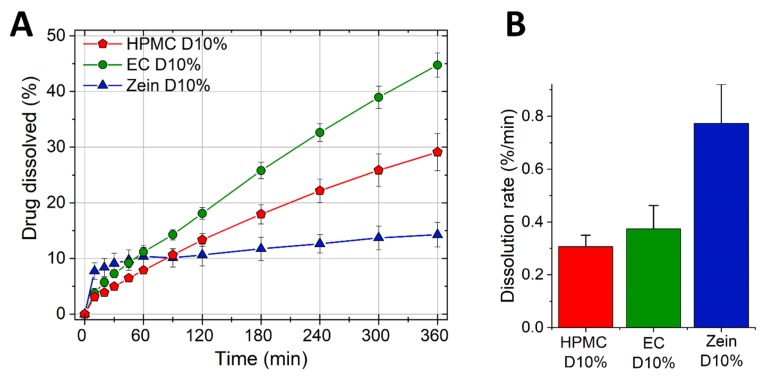
Dissolution profiles of HPMC, EC, and zein tablets containing 10% propranolol HCl. (**A**) Percentage of drug release (mean ± SD, *n* = 3) as a function of time. (**B**) Drug burst (mean ± SD, *n* = 3) measured as the dissolution rate in the first 10 min of the test.

We then incorporated 10% propranolol HCl into the formulations (Table 1, Table 2 and Table 3) and the drug release was determined as seen in Figure 2A.

The drug burst measured as the dissolution rate in the first 10 min of the test is presented in Figure 2B. Figure 2A shows a gradual and continuous release of drug from HPMC and EC tablets. On the contrary, zein matrices had an initial drug burst in the first hour, followed by a very slow release thereafter. In the case of the two swelling polymers, i.e., HPMC and zein, the release profiles (Figure 2) are a mirror image of the swelling results seen in Figure 1. As explained earlier, HPMC being highly hydrophilic gels rapidly on the surface hindering further swelling and drug burst. Then, the gel provides a diffusion barrier which enables a slow and continuous water diffusion into the core (swelling) and drug diffusion out of the core (release). In the case of zein, water diffuses in and drug diffuses out of the tablet more rapidly at first, as shown by the greater initial swelling and drug release compared to HPMC. However, the swelling of zein was later sufficient to close-up the pores and create a strong barrier to further water ingress (and swelling) and drug release. The ability of zein to hydrate, coalesce and fill-up the voids in the matrix has been previously described [10,34]. Once the polymer hydration is complete, the diffusion barrier offered by the swollen zein is greater than that of HPMC, as demonstrated by the much slower swelling and drug release rates of zein compared to HPMC.

The burst release observed with zein here has been documented in other studies with tablets and capsules, where zein and drug were present as physical mixtures and pores were present in the matrix [7,10]. However, in the case of hot-melted extruded caplets, characterized by a continuous and less porous matrix and by a more intimate mixing between drug and polymer, the drug burst was not observed [11,12].

### 3.2. Swelling and Drug Release of Matrices Containing Diluents

#### 3.2.1. Swelling

**Figure 3:**
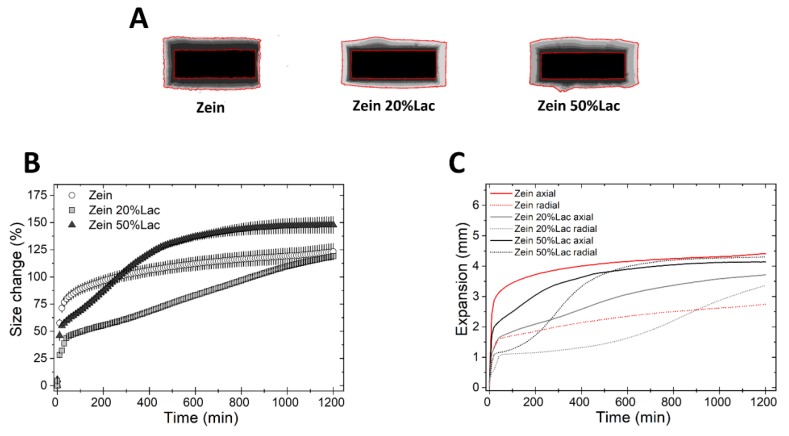
Swelling of tablets made of binary mixtures of zein and lactose (20% and 50% *w*/*w*). (**A**) Sum of Z-stack of images. The two red lines are the outlines of the tablet at time 0 and at 20 h. (**B**) Swelling measured as the change of the projected area of tablets (mean ± SD, *n* = 3) as a function of time. (**C**) Axial and radial swelling of tablets (mean ± SD, *n* = 3) as a function of time.

We then studied the swelling and drug release from tablets made of binary mixtures of the three polymers and diluents. First, we investigated the effect of lactose, a soluble non-swellable diluent [21], on matrix swelling (Figure 3). Compared to tablets of pure zein, zein tablets containing 20 and 50% of lactose (Table 1) swelled less rapidly in the first 100 min of the test, but more rapidly thereafter, as seen in Figure 3B. Moreover, the presence of lactose in the matrix resulted in a simultaneous decrease in the axial swelling and an increase in the radial swelling (Figure 3C). The same experiment was conducted using HPMC and EC, as controls. The incorporation of lactose in HPMC-based matrices did not cause substantial changes in the swelling kinetics (Figure S1), in agreement with the work of Gao et al. [35]. Similarly, EC matrices containing lactose showed the same extent of swelling of pure EC tablets.

**Figure 4:**
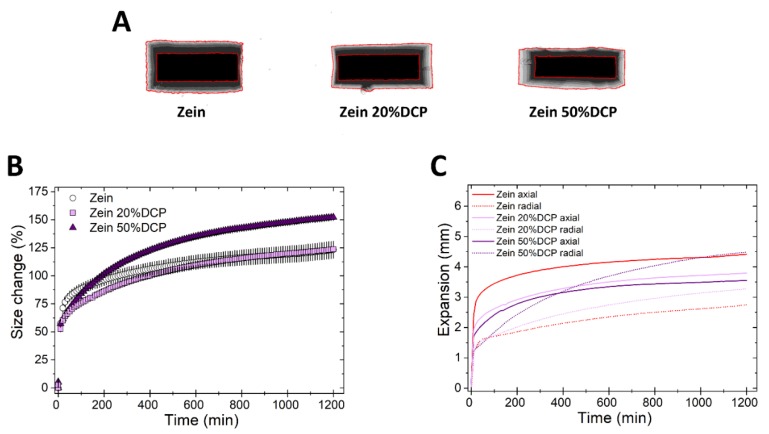
Swelling of tablets made of binary mixtures of zein and DCP (20% and 50% *w*/*w*). (**A**) Sum of Z-stack of images. The two red lines are the outlines of the tablet at time 0 and at 20 h. (**B**) Swelling measured as the change of the projected area of tablets (mean ± SD, *n* = 3) as a function of time. (**C**) Axial and radial swelling of tablets (mean ± SD, *n* = 3) as a function of time.

Next, we studied the swelling of matrices upon the incorporation of DCP, an insoluble non-swellable hydrophilic diluent [21], at two concentrations (20% and 50% *w*/*w*). The addition of DCP did not influence the kinetics of volumetric swelling of zein matrices at low concentration, yet an increase in rate and extent of size change was observed at the higher DCP concentration (Figure 4A,B). Swelling in the radial direction was more favored at increasing concentrations of DCP, as can be seen in Figure 4A,C. This latter result was also observed upon the incorporation of lactose. Interestingly, HPMC-based tablets showed the same, whereby the axial swelling decreased, and the radial swelling increased at increasing the DCP concentration (Figure S2). This change in the direction of swelling of zein and HPMC- based matrices caused by DCP and lactose can possibly be attributed to an increase in the fragmentation behavior of the powder (typical of both DCP and lactose [36,37,38]) and thus a decrease in plastic deformation upon compression. The reduced plastic deformation means that, upon exposure to fluids, the tablets relaxation in the axial direction decreases. Simultaneously, the wicking effect of the hydrophilic DCP and lactose can possibly favor hydration and thus omni-directional swelling of the polymer particles.

**Figure 5:**
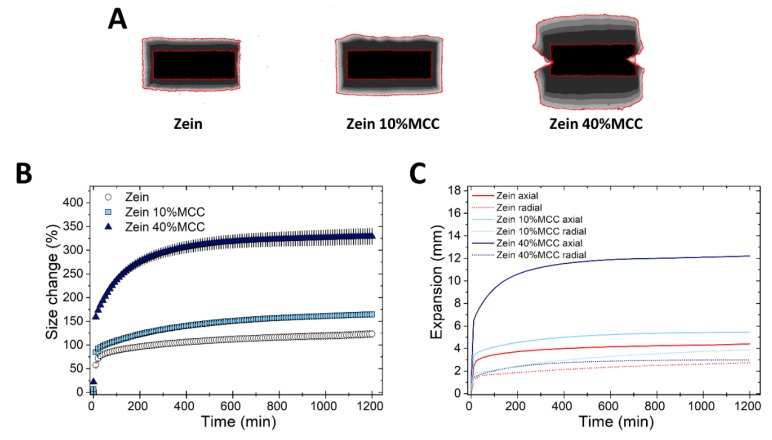
Swelling of tablets made of binary mixtures of zein and MCC (10% and 40% *w*/*w*). (**A**) Sum of Z-stack of images. The two red lines are the outlines of the tablet at time 0 and at 20 h. (**B**) Swelling measured as the change of the projected area of tablets (mean ± SD, *n* = 3) as a function of time. (**C**) Axial and radial swelling of tablets (mean ± SD, *n* = 3) as a function of time.

Finally, the swelling of zein tablets in the presence of MCC, a hydrophilic, swellable, yet insoluble diluent [39], was studied at two concentrations (10% and 40% *w*/*w*). Figure 5 shows that while zein 10% MCC tablets swelled only slightly more than the pure zein tablets, zein 40%MCC tablets had an approximately three-fold increase in size expansion, compared to the pure tablets. This increase in size expansion caused by MCC was only in the axial and not in the radial direction (Figure 5B). The Z-stack of the swelling of zein 40% MCC tablets reveals that matrices deformed into an “hourglass” shape, yet the integrity of the monolith was maintained. The “hour-glass” shape can be ascribed to the release of the stored energy of compression upon hydration of the plastic MCC. This shape of expansion has been previously documented for immediate release tablets containing highly plastic, so-called shape-recovery disintegrants (e.g., crospovidone) [23,40]. Since the density of a flat-shaped tablet is greater at the edges [41,42], the release of energy of the compressed MCC particles upon hydration is greater at the edges than in other areas of the tablet, ultimately resulting in the formation of this “hour-glass” shape.

In the case of HPMC, the incorporation of MCC within the matrix did not influence the swelling kinetics, as shown in Figure S3. On the contrary, the insoluble EC matrices maintained their integrity only at the lower MCC concentration (i.e., 10%), yet the tablets broke-up rapidly at the higher concentration (i.e., 40%), probably due to the disintegration action of MCC [21]. Interestingly, the effect of MCC on the swelling of zein, HPMC and EC matrices is completely different. On one side, both HPMC and MCC, being viscoelastic materials which undergo plastic deformation [43], promote axial swelling. Moreover, the incorporation of MCC into HPMC tablets even at concentrations as high as 40% does not affect the swelling and integrity of the matrix, possibly due to the high gelling ability of HPMC. On the opposite side tablets of EC, stiffer and practically non-swellable polymer, disintegrate at high (40%) MCC concentration. Zein has intermediate swelling behavior between that of HPMC and EC, and consequently, zein matrices show an increased axial swelling, but still maintained the matrix integrity in the presence of MCC. In agreement with the results obtained here, we had previously shown that SMCC (98% MCC and 2% colloidal silico dioxide) swells mainly axially and thus amplifies the axial swelling when it is incorporated into zein matrices [13].

#### 3.2.2. Drug Release

**Figure 6:**
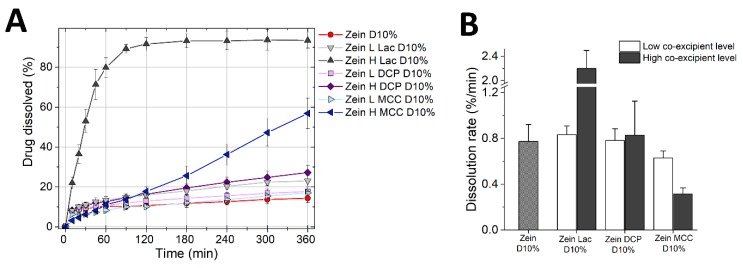
Dissolution profiles of tablets made of ternary mixtures of zein, diluent (lactose, DCP or MCC) and 10% propranolol HCl. (**A**) Percentage of drug release (mean ± SD, *n* = 3) as a function of time. (**B**) Drug burst (mean ± SD, *n* = 3) measured as the dissolution rate in the first 10 min of the test.

Next, we studied the drug release from tablets containing ternary mixtures of zein, diluents, and propranolol HCl (Figure 6).

It is worth noting that ratios of zein/diluents in these formulations were kept the same as those of tablets used in the previous swelling tests. Figure 6A shows that the drug release from zein tablets containing the lower concentration of diluents, i.e., lactose, DCP, and MCC, was similar to that obtained with the binary mixture of zein and drug. However, different drug release kinetics were obtained when the additives were incorporated in high concentration. The drug release from zein formulation containing 45% lactose (zein H Lac D10%, Table 1) was rapid, possibly due to the dissolution of the lactose in the matrix and the consequent formation of pores, which facilitated water and drug diffusion. On the contrary, zein H DCP D10% tablets showed only a slightly faster drug release compared to the zein/drug tablets. DCP is hydrophilic in nature and could favor water penetration into the tablet, yet being insoluble, DCP remained trapped within the matrix, creating a barrier to rapid drug release. The release profile obtained here is similar to that of tablets containing, zein, DCP, and theophylline studied by Georget et al. [7]. Finally, the drug release from zein H MCC D10% tablets was more rapid than that of zein/drug tablets, with >50% of propranolol released in 6 h, and drug release was nearly linear over the test duration. Figure 5B demonstrated that MCC can greatly increase the macroscopic swelling of the zein matrices. Therefore, it could be suggested that the faster drug release from zein/MCC tablets is due to an increase of polymer relaxation of zein and thus a reduction of the diffusion barrier of the matrix.

Figure 6B highlights the initial release rate from the same formulations. It is evident that lactose acts as a pore former and thus increased the drug burst from the matrix in a concentration-dependent manner. DCP did not seem to have a major effect on the drug burst. Surprisingly, the presence of MCC in zein matrices suppressed the drug burst: At the higher MCC concentration, the initial drug release rate was approximately 3-times lower than that from zein/drug tablets. This is a paradox, especially because MCC is a disintegrant, and can be explained by referring to the swelling studies. MCC is hydrophilic and thus absorbed a large amount of water in the first instant of exposure to fluids, favoring hydration and swelling of zein (Figure 5B). The voids through which the drug could leach out were therefore closed by the rapid uncoiling of zein polymer, decreasing the drug burst. Interestingly, the swelling/polymer uncoiling can, on one side close the pores responsible for the premature drug release, and on the other side, provide the matrix relaxation necessary to promote continuous drug release on the long term.

Overall, zein only tablets provided an initial burst and only very slow release thereafter (Figure 2), offering little advantage for most controlled release applications. The incorporation of MCC in zein matrices could provide the dual benefit of suppressing the initial drug burst and speeding up the drug release over time. In other words, the addition of MCC gives a nearly constant drug release, which is the target of many sustained-release technologies [44].

### 3.3. Swelling and Drug Release of Matrices containing PVP and SLS

#### 3.3.1. Swelling

**Figure 7:**
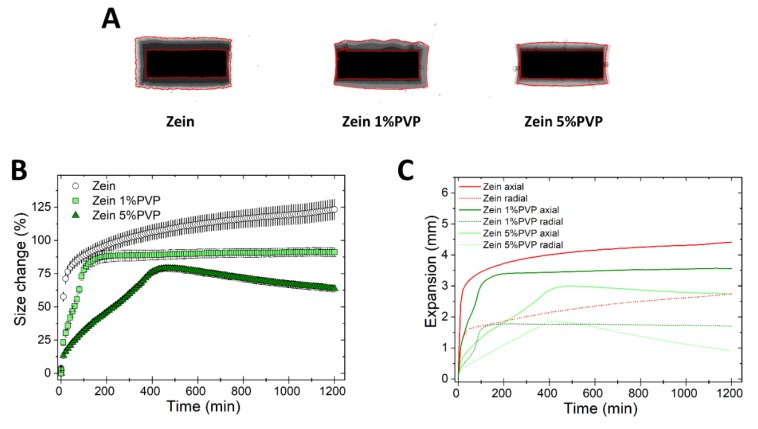
Swelling of tablets made of binary mixtures of zein and PVP (1% and 5% *w*/*w*). (**A**) Sum of Z-stack of images. The two red lines are the outlines of the tablet at time 0 and at 20 h. (**B**) Swelling measured as the change of the projected area of tablets (mean ± SD, *n* = 3) as a function of time. (**C**) Axial and radial swelling of tablets (mean ± SD, *n* = 3) as a function of time.

To understand the effect of binders on the swelling of zein matrices, tablets of binary mixtures of zein and either 1% or 5% PVP K30 were prepared. The swelling behavior of zein/PVP tablets is shown in Figure 7.

Figure 7A shows that the tablets containing PVP swelled less than tablets of pure zein, possibly because the binder hydration created a further barrier to water ingress and matrix swelling. Zein 5% PVP tablets showed peculiar profiles (Figure 7B,C), whereby the tablets swelled more slowly than the pure zein tablets, and after reaching a swelling maximum at approximately 500 min, the matrix size started to decline over time both in the axial and radial directions. It can be hypothesized that the binder hydration might have increased the adhesion between adjacent particles in the matrix, leading to a resistance to size expansion, and even to a modest matrix shrinkage at time >500 min. The formulation zein 1% PVP showed an intermediate swelling behavior between zein and zein 5% PVP. In comparison to zein-based matrices, the swelling of HPMC matrices was not influenced by the presence of PVP (Figure S4).

**Figure 8:**
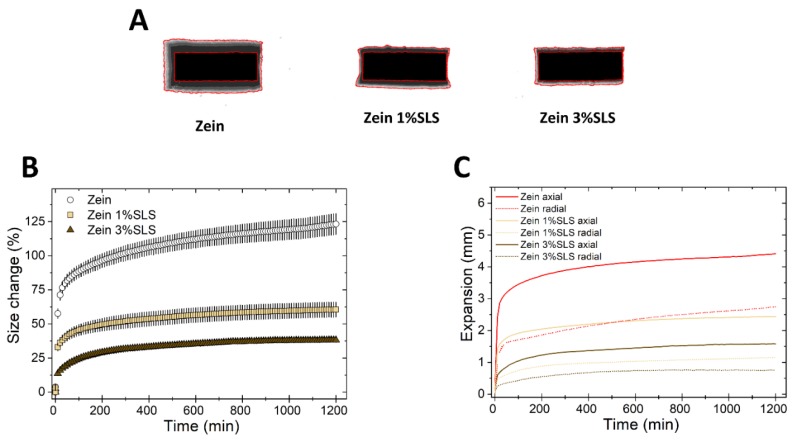
Swelling of tablets made of binary mixtures of zein and SLS (1% and 3% *w*/*w*). (**A**) Sum of Z-stack of images. The two red lines are the outlines of the tablet at time 0 and at 20 h. (**B**) Swelling measured as the change of the projected area of tablets (mean ± SD, *n* = 3) as a function of time. (**C**) Axial and radial swelling of tablets (mean ± SD, *n* = 3) as a function of time.

Next, we evaluated the effect of the ionic surfactant SLS on the swelling of zein. Figure 8 shows that the swelling of zein matrices was greatly suppressed by increasing the concentration of SLS in the matrix. This is due to the zein/SLS interaction and also, possibly, to the denaturing effect of SLS on zein. It has been previously shown that the interaction of SLS in solution and zein is complex [45,46]: At low SLS concentration, the negatively charged surfactant can be electrostatically attracted to the positively charged head groups of zein. At higher SLS concentrations, when all the positive charges on zein become neutralized, the repulsion between the negative charges of the surfactant and polymer begins, driving both zein unfolding and interaction between the hydrophobic portions of surfactant and polymer. In comparison to zein, the swelling of HPMC was not influenced by the presence of SLS in the matrix, whereas EC matrices had an increased axial expansion in the presence of the surfactant (Figure S5). In conclusion, SLS showed a clearly distinctive effect on the swelling of each of the three polymers.

#### 3.3.2. Drug Release

The drug release kinetics, as well as the drug release rate in the first 10 min of zein/PVP and zein/SLS tablets containing 10% propranolol HCl, were similar to those obtained with zein/propranolol HCl tablets (Figure S6). It can be hypothesized that, in tablets containing PVP and SLS, factors that promoted the drug release and those that hindered the release balanced each other. For instance, PVP reduced tablet swelling and thus hindered the matrix relaxation and consequently, the drug release. However, faster water diffusion into the matrix might have been promoted by the low viscosity hydrophilic PVP. These two opposing factors might have balanced each other, keeping the release profile similar to that of zein only tablets.

In the case of SLS, on one hand, this wetting agent is thought to promote liquid penetration into tablets by decreasing the interfacial tension with the media. On the other hand, this anionic surfactant could interact with zein [45,46], suppressing the swelling (Figure 8) and could also form a complex with the cationic drug, propranolol HCl. The complex SLS-propranolol HCl is likely to be less soluble than the free drug [47]. As a result of these opposing factors, the release of propranolol HCl from zein/SLS matrices remains unchanged compared to the pure zein and propranolol HCl tablets.

## 4. Conclusions

The image analysis technique adopted in this work enabled continuous monitoring of tablet swelling over a 20-h test period and brought about even small differences in swelling between formulations. Considering that the USB microscope used for collecting the images can be purchased for only $35 and that the image analysis software is open access, we anticipate that this technique could be adopted in the future studies, as a direct readout of swelling of controlled release polymer matrices.

We have shown in this work that tablets of amphiphilic zein have different swelling and release patterns compared to HPMC and EC tablets. Zein is peculiar in the mode of swelling and drug release, which are fast initially, but then rapidly reach a plateau. Although the addition of DCP, PVP, and SLS to zein matrices could influence the kinetics of axial and radial swelling, no significant impact on drug release was observed. On the contrary, the incorporation of both lactose and MCC had a major impact on drug release kinetics. Interestingly, the addition of MCC to zein matrix could provide a more constant drug release compared to pure zein matrix, thus offering a great advantage for designing efficient controlled drug delivery devices.

In conclusion, this work has provided information on the effect of additives on the performance of zein tablets, which will be useful in formulation development. Possible zein-co-excipient interactions were only hypothesized based on their physico-chemical properties, yet the investigation of the specific molecular interactions at the zein/co-excipient interface is beyond the aim of this work and could be undertaken in future investigations. This study has also identified that the swelling behavior of zein matrices both alone and in combination with additives is clearly different from that of typical hydrophilic and insoluble polymers, such as HPMC and EC, respectively.

## Figures and Tables

**Table 1:** Composition of zein-based matrix formulations.

	Abbreviation		Components (% *w*/*w*)						
		Propranolol HCl	Zein	Lac	DCP	MCC	PVP	SLS	Magnesium Stearate
	Zein	-	99.5	-	-	-	-	-	0.5
	Zein 10%D	10.0	89.5	-	-	-	-	-	0.5
Binary mixtures	Zein 20%Lac	-	79.5	20.0	-	-	-	-	0.5
	Zein 50%Lac	-	49.5	50.0	-	-	-	-	0.5
	Zein 20%DCP	-	79.5	-	20.0	-	-	-	0.5
	Zein 50%DCP	-	49.5	-	50.0	-	-	-	0.5
	Zein 10%MCC	-	89.5	-	-	10.0	-	-	0.5
	Zein 40%MCC	-	59.5	-	-	40.0	-	-	0.5
	Zein 1%PVP	-	98.5	-	-	-	1.0	-	0.5
	Zein 5%PVP	-	94.5	-	-	-	5.0	-	0.5
	Zein 1%SLS	-	98.5	-	-	-	-	1.0	0.5
	Zein 3%SLS	-	96.5	-	-	-	-	3.0	0.5
Ternary mixtures	Zein L Lac 10%D	10.0	71.5	18.0	-	-	-	-	0.5
	Zein H Lac 10%D	10.0	44.52	44.98	-	-	-	-	0.5
	Zein L DCP 10%D	10.0	71.5	-	18.0	-	-	-	0.5
	Zein H DCP 10%D	10.0	44.52	-	44.98	-	-	-	0.5
	Zein L MCC 10%D	10.0	80.5	-	-	9.0	-	-	0.5
	Zein H MCC 10%D	10.0	53.52	-	-	35.98	-	-	0.5
	Zein L PVP 10%D	10.0	88.60	-	-	-	0.9	-	0.5
	Zein H PVP 10%D	10.0	85.0	-	-	-	4.5	-	0.5
	Zein L SLS 10%D	10.0	88.6	-	-	-	-	0.9	0.5
	Zein H SLS 10%D	10.0	86.80	-	-	-	-	2.7	0.5

**Table 2:** Composition of HPMC-based matrix formulations.

	Abbreviation	Components (% *w*/*w*)								
		Propranolol HCl	HPMC	Lac	DCP	MCC	PVP	SLS	TiO_2_	Magnesium Stearate
	HPMC	-	99.0	-	-	-	-	-	0.5	0.5
	HPMC 10%D	10	89.0	-	-	-	-	-	0.5	0.5
Binary mixtures	HPMC 20%Lac	-	79.0	20.0	-	-	-	-	0.5	0.5
	HPMC 50%Lac	-	49.0	50.0	-	-	-	-	0.5	0.5
	HPMC 20%DCP	-	79.0	-	20.0	-	-	-	0.5	0.5
	HPMC 50%DCP	-	49.0	-	50.0	-	-	-	0.5	0.5
	HPMC 10%MCC	-	89.0	-	-	10.0	-	-	0.5	0.5
	HPMC 40%MCC	-	59.0	-	-	40.0	-	-	0.5	0.5
	HPMC 1%PVP	-	98.0	-	-	-	1.0	-	0.5	0.5
	HPMC 5%PVP	-	94.0	-	-	-	5.0	-	0.5	0.5
	HPMC 1%SLS	-	98.0	-	-	-	-	1.0	0.5	0.5
	HPMC 3%SLS	-	96.0	-	-	-	-	3.0	0.5	0.5

**Table 3:** Composition of EC-based matrix formulations.

	Abbreviation	Components (% *w*/*w*)							
		Propranolol HCl	EC	Lac	DCP	MCC	PVP	SLS	Magnesium Stearate
	EC	-	99.5	-	-	-	-	-	0.5
	EC 10%D	10	89.5	-	-	-	-	-	0.5
Binary mixtures	EC 20%Lac	-	79.5	20.0	-	-	-	-	0.5
	EC 50%Lac	-	49.5	50.0	-	-	-	-	0.5
	EC 20%DCP	-	79.5	-	20.0	-	-	-	0.5
	EC 50%DCP	-	49.5	-	50.0	-	-	-	0.5
	EC 10%MCC	-	89.5	-	-	10.0	-	-	0.5
	EC 40%MCC	-	59.5	-	-	40.0	-	-	0.5
	EC 1%PVP	-	98.5	-	-	-	1.0	-	0.5
	EC 5%PVP	-	94.5	-	-	-	5.0	-	0.5
	EC 1%SLS	-	98.5	-	-	-	-	1.0	0.5
	EC 3%SLS	-	96.5	-	-	-	-	3.0	0.5

## Supplementary Materials

The following are available online at https://www.mdpi.com/1999-4923/11/10/513/s1, Supplementary information, Video S1: time-lapse of the swelling of HPMC, EC and zein tablets.
