# Supplementary material for: Swelling of Zein Matrix Tablets Benchmarked against HPMC and Ethylcellulose: Challenging the Matrix Performance by the Addition of Co-Excipients
Source: Pharmaceutics. 2019 Oct 4;11(10):513. doi: 10.3390/pharmaceutics11100513 (PMC6836314; doi:10.3390/pharmaceutics11100513)
Supplement: Supplementary file 1 [file pharmaceutics-11-00513-s001.zip › pharmaceutics-586746-supplementary/pharmaceutics-586746-supplementary.pdf]

Supplementary Materials: Swelling of Zein Matrix Tablets Benchmarked against HPMC and Ethylcellulose: Challenging the Matrix Performance by the Addition of Co-Excipients

Alberto Berardi, Safwan Abdel Rahim, Lorina Bisharat and Marco Cespi

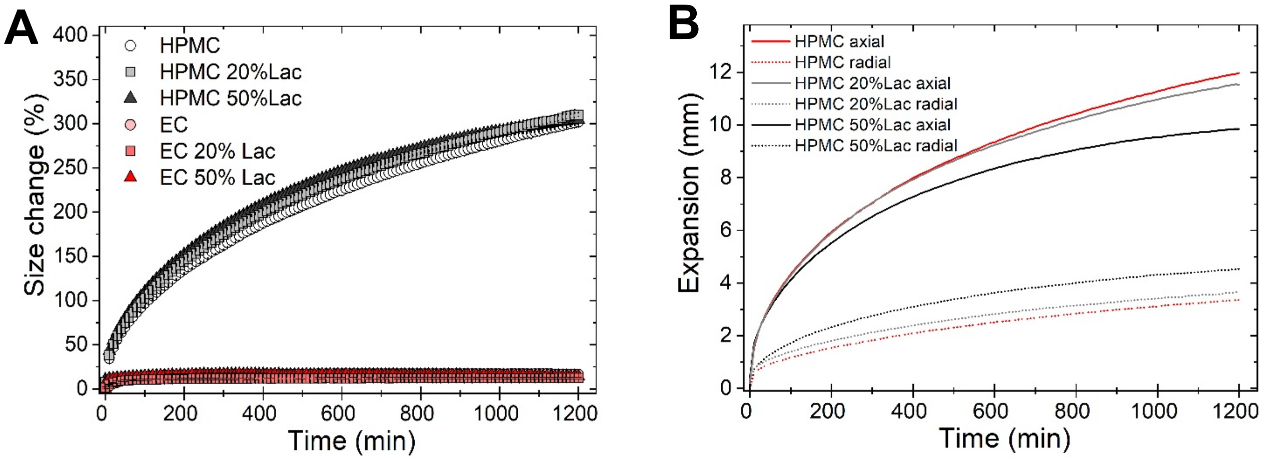

Figure 1. Swelling of tablets made of binary mixtures of HPMC or EC with lactose (20% and 50% w/w). Swelling measured as change of projected area of tablets (mean ± SD, n = 3) as a function of time. (C) Axial and radial swelling of HPMC tablets (mean ± SD, n = 3) as a function of time. Data of axial and radial expansion of EC tablets were not reported because values of swelling remained negligible at all conditions, as it can also be noticed in panel A. .

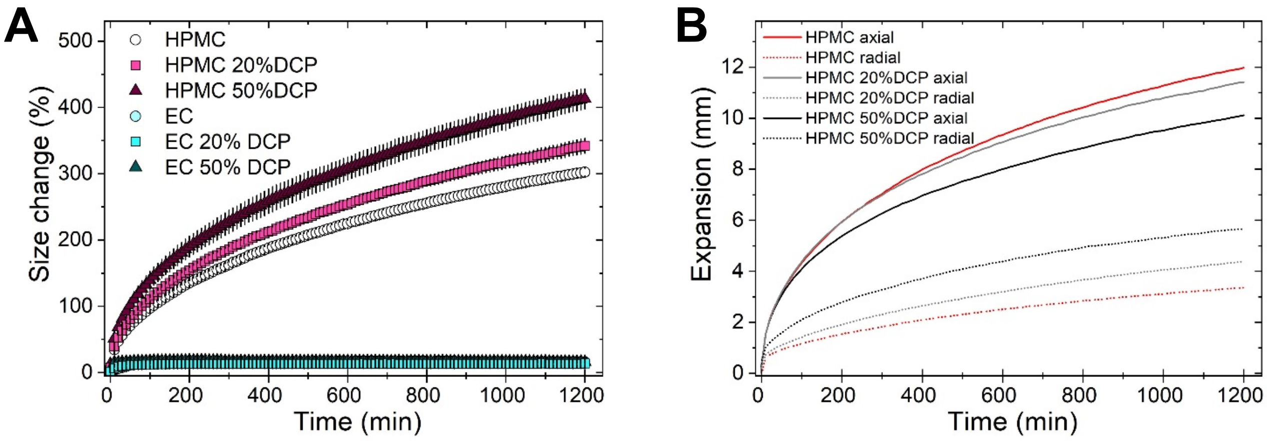

Figure 2. Swelling of tablets made of binary mixtures of HPMC or EC with DCP (20% and 50% w/w). Swelling measured as change of projected area of tablets (mean ± SD, n = 3) as a function of time. (C) Axial and radial swelling of HPMC tablets (mean ± SD, n = 3) as a function of time. Data of axial and radial expansion of EC tablets were not reported because values of swelling remained negligible at all conditions, as it can also be noticed in panel A. .

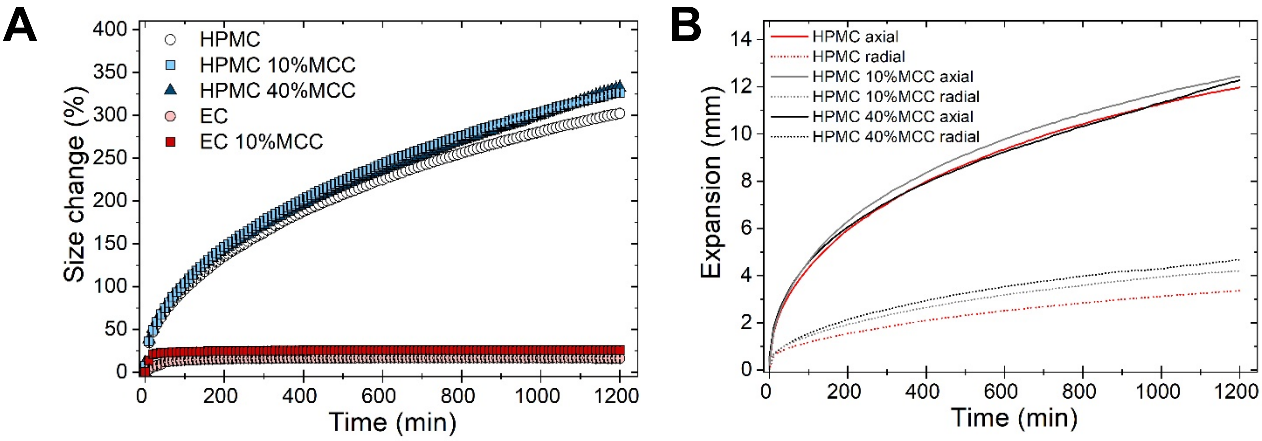

Figure 3. Swelling of tablets made of binary mixtures of HPMC or EC with MCC (10% and 40% w/w). Swelling measured as change of projected area of tablets (mean ± SD, n = 3) as a function of time. (C) Axial and radial swelling of HPMC tablets (mean ± SD, n = 3) as a function of time. Data of axial and radial expansion of EC tablets were not reported because values of swelling remained negligible at all conditions, as it can also be noticed in panel A. .

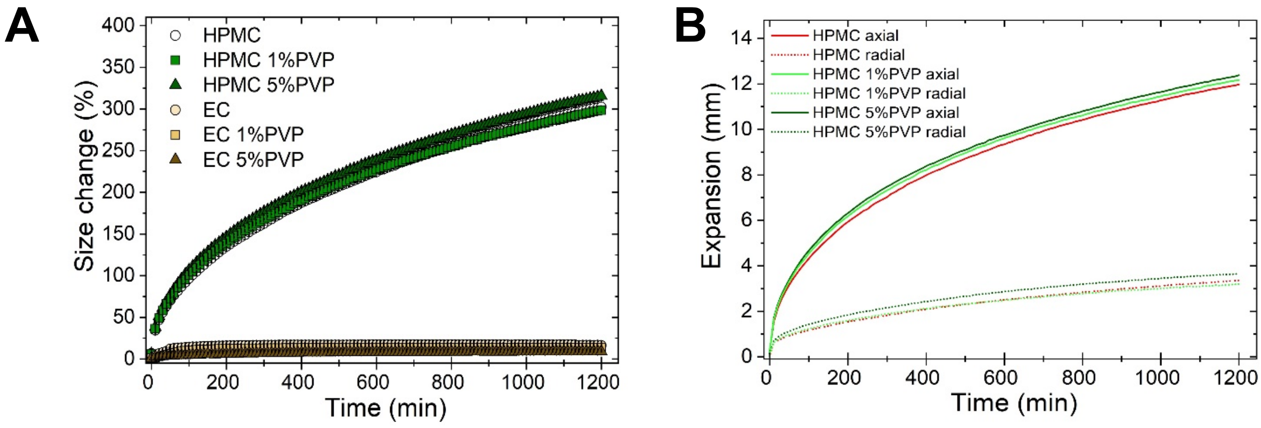

Figure 4. Swelling of tablets made of binary mixtures of HPMC or EC with PVP (1% and 5% w/w). Swelling measured as change of projected area of tablets (mean ± SD, n = 3) as a function of time. (C) Axial and radial swelling of HPMC tablets (mean ± SD, n = 3) as a function of time. Data of axial and radial expansion of EC tablets were not reported because values of swelling remained negligible at all conditions, as it can also be noticed in panel A. .

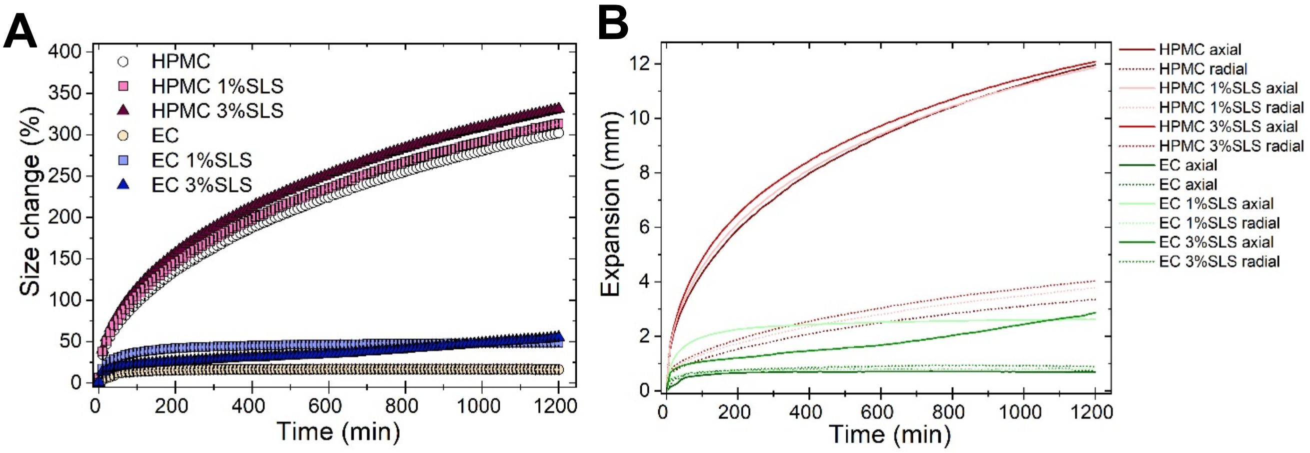

Figure S5. Swelling of tablets made of binary mixtures of HPMC or EC with SLS (1% and 3% w/w). Swelling measured as change of projected area of tablets (mean ± SD, n = 3) as a function of time. (C) Axial and radial swelling of tablets (mean ± SD, n = 3) as a function of time. It can be noticed that, in presence of SLS, EC swelled axially with the formation of an apex at either the bottom or top surface of the otherwise flat tablet. .

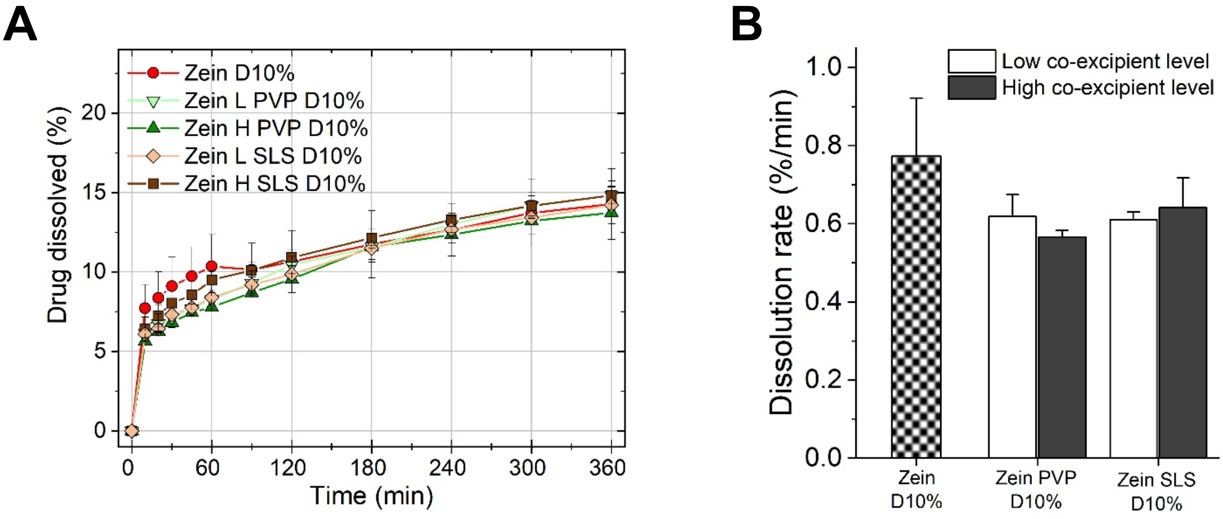

Figure S6. Dissolution profiles of tablets made of ternary mixtures of zein, PVP or SLS and 10% propranolol HCl. (A) Percentage of drug release (mean ± SD, n = 3) as a function of time. (B) Drug burst (mean ± SD, n = 3) measured as the dissolution rate in the first 10 minutes of the test.
